# Correlation of Serum Vitamin D and High-Density Lipoprotein (HDL) Cholesterol Levels With Disease Activity in Rheumatoid Arthritis: A Single-Center Experience From Eastern India

**DOI:** 10.7759/cureus.69333

**Published:** 2024-09-13

**Authors:** Cankatika Choudhury, Akhil Sahib, Partha Karmakar, Suvrendu Kar

**Affiliations:** 1 Neurology, Govind Ballabh Pant Institute of Postgraduate Medical Education and Research, New Delhi, IND; 2 General Medicine, R. G. Kar Medical College and Hospital, Kolkata, IND; 3 Endocrinology and Metabolism, Institute of Post Graduate Medical Education & Research, Kolkata, IND

**Keywords:** disease activity, high density lipoprotein, rheumatoid arthritis, systemic inflammation, vitamin d

## Abstract

Background

Rheumatoid arthritis (RA) is a progressive, symmetric, and erosive polyarthritis with a variety of extraarticular manifestations such as mononeuritis multiplex, central nervous system vasculitis, Felty’s syndrome, dyslipidemia, carditis, and interstitial lung disease. Vitamin D plays a role in both adaptive and innate immunity, and its deficiency leads to the development of many autoimmune disorders. Additionally, RA patients have a lipid paradox consisting particularly of dysfunctional and low levels of high-density lipoprotein (HDL) with reduced low-density lipoprotein lowering effect, which increases cardiovascular morbidity and potentiates widespread systemic inflammation. Both are modifiable risk factors. Although there are numerous studies on vitamin D and HDL cholesterol in disease progression in RA, there is sparse literature from India studying both these factors in combination. In this study, we tried to establish the correlation of serum vitamin D and HDL cholesterol levels, if any, with disease activity using the Disease Activity Score 28 Erythrocyte Sedimentation Rate (DAS28 ESR) score.

Methods

A descriptive cross-sectional study comprising 80 patients was conducted at a tertiary care center in Eastern India over 12 months. Newly diagnosed RA patients aged >17 years satisfying the diagnostic criteria were included. Serum vitamin D level and HDL cholesterol were measured. Then, the DAS28 ESR score was calculated, and a correlation was looked for between serum vitamin D and HDL cholesterol.

Results

Patients aged 35-43 years accounted for 32 (42.5%) of participants, of whom 58 (72.5%) were females. Almost half, 38 (47.5%), had vitamin D deficiency. The mean vitamin D level was 22.988 ± 10.01 ng/ml. The mean HDL cholesterol level was 42.3 ± 7.23 mg/dl. The mean DAS28-ESR score was 3.81 ± 1.19. A statistically significant inverse correlation was found between vitamin D levels and DAS28 ESR score (p -0.0003) and HDL (p -0.000349).

Conclusions

Vitamin D deficiency and low HDL cholesterol levels are more common in RA patients. These factors may contribute to increased disease activity. Both are treatable factors in addition to conventional therapies.

## Introduction

Rheumatoid arthritis (RA) is the most common cause of progressive, inflammatory, symmetrical, and erosive arthritis [1]. The reported prevalence of RA in India is just higher with respect to the global prevalence [1,2]. It is two to three times more common in women, and the average age of onset is 55 years [1]. It has multisystem manifestations such as mononeuritis multiplex; secondary vasculitis; cervical myelopathy secondary to atlantoaxial instability; and hematological malignancies like lymphoma, pericarditis, pulmonary nodules, and interstitial lung disease, to name a few. The outward appearance of joints does not always correlate with the degree of pain and synovitis. In terms of the chronological order of joint involvement, proximal interphalangeal joints, followed in sequence by the metacarpophalangeal joints, wrist, elbow, shoulder, knee, ankle, subtalar, and metatarsophalangeal joints, are commonly seen. It not only has high morbidity associated with joint deformity and systemic complications but also carries a high risk of mortality due to increased atherosclerotic burden secondary to widespread inflammation [3]. Vitamin D plays a key role in adaptive and innate immunity and has been shown to function as an anti-inflammatory in autoimmune disorders such as RA, insulin-dependent diabetes mellitus, systemic lupus erythematosus, multiple sclerosis, Hashimoto’s thyroiditis, ankylosing spondylitis, and inflammatory bowel disease [4,5]. Its active form, 1,25-dihydroxyvitamin D or calcitriol, acts through vitamin D receptor (VDR) on the surface of T and B cells. In health, it stimulates the Th2 pathway leading to the production of anti-inflammatory cytokines, and it also activates T-regulatory cells, maintaining a state of immune tolerance. It also suppresses B cell differentiation and proliferation. Its deficiency leads to activation of the Th1 pathway, leading to unregulated systemic inflammation and destruction through pro-inflammatory mediators such as IL-2, interferon-γ, and tumor necrosis factor-α [4,5]. VDRs are also found on the surface of synovial macrophages and chondrocytes, particularly at sites of erosive destruction [4,5]. This inflammatory state is associated with qualitative as well as quantitative changes in the anti-inflammatory and atheroprotective functions of high-density lipoprotein (HDL) cholesterol, which is now better known as “pro-inflammatory HDL” [6]. Further, the cholesterol efflux capacity (CEC) of HDL and its low-density lipoprotein (LDL) lowering effect become impaired, thereby increasing cardiovascular risk by twofold despite having a seemingly less atherogenic profile. This is known as the lipid paradox, wherein the levels of total cholesterol and LDL cholesterol are low and HDL cholesterol is not only low but highly dysfunctional [6]. Low serum levels of vitamin D and HDL are modifiable and treatable risk factors. Also, there are limited studies assessing both of these factors together in RA progression. Hence, we tried to establish any correlation between vitamin D and HDL cholesterol levels with disease activity in RA using the Disease Activity Score 28 Erythrocyte Sedimentation Rate (DAS28 ESR) score [4,5,7].

The aim was to determine the relationship of serum vitamin D and HDL cholesterol levels with disease activity in newly diagnosed RA. The primary objective was to study the level of serum vitamin D and HDL cholesterol in RA and to find its correlation with the DAS28 ESR score.

## Materials and methods

Study design and population

This was a descriptive cross-sectional study conducted in the Department of General Medicine of R. G. Kar Medical College and Hospital in Kolkata, India, over 12 months. A total of 80 patients were included. The first case was selected by random sampling by a two-digit random number table, and the second case was selected by systematic random sampling. Patients with newly diagnosed RA aged >17 years according to the American College of Rheumatology/European League Against Rheumatism Criteria 2010 were included in the study [8]. Patients with RA who were already on treatment with disease-modifying antirheumatic drugs/biological response modifiers; those having chronic renal failure, diabetes mellitus, preexisting cardiac or respiratory disorders, coexistent systemic lupus erythematosus, Sjogren’s syndrome, systemic sclerosis, or mixed connective tissue disorder; and those on enzyme-inducing drugs, on calcium or vitamin D supplements, on lipid-lowering drugs (statins/fibrates/niacin), or on beta-blockers were excluded.

Data collection

Data were collected as per the pre-designed patient proforma. Detailed history included age of onset, pattern of joint involvement, progression of disease, presence of pain, and swelling. Serum 25-hydroxyvitamin D level required about 10 ml of fasting blood sample and was tested using the ELISA kit in the Biotek ELX-800 autoanalyzer. The researchers measured 25-OH vitamin D because it has a longer half-life. Serum lipid levels, including HDL cholesterol, were obtained after nine to 12 hours of fasting and were assessed using a Hitachi 912 analyzer (Roche Diagnostics, Germany). A normal value of 25-OH vitamin D was defined as 30-40 ng/ml, vitamin D sufficiency: ≥30ng/ml, vitamin D deficiency: ≤20ng/ml, and vitamin D insufficiency: 21-29 ng/ml. Normal HDL cholesterol level for adults is 40-60 mg/dl. Other investigations were erythrocyte sedimentation rate (ESR), rheumatoid factor (RF) by nephelometric assay (cutoff >20), anti-citrullinated cyclic peptide antibody (anti-CCP) titers using latex agglutination method (cutoff >1:40), complete blood count, liver function test, renal function test, and serum calcium. Determined values of vitamin D and HDL were analyzed and correlated with the DAS28 ESR.

DAS28 ESR is a summation of 28 swollen and tender joints, general health, and ESR. The score ranges between 0 and 9.4. The patient’s general health is assessed using the Visual Analogue Scale (VAS), which is a 100-mm scale where 0 = best response and 100 = worst. The higher the score, the greater the disease activity. Disease activity was calculated using the DAS28 ESR score using the formula DAS28 = 0.56√(28TJC) + 0.28√(28SJC) + 0.70 Ln (ESR) + 0.014 VAS. The components of the mathematical formula are as follows: TJC is tender joint count, SJC is swollen joint count, Ln is log, and VAS is Visual Analogue Scale. Disease severity was then categorized according to the score obtained as follows: DAS28 <2.6 suggestive of remission, 2.6 to ≤3.2 suggestive of low disease activity, >3.2 to ≤5.1 suggestive of moderate disease activity, and DAS28 >5.1 suggestive of high disease activity.

Ethical consideration

Permission was taken from the Institutional Ethics Committee of R. G. Kar Medical College and Hospital (approval number 3560/c of 13/01/15). Written informed consent was taken from each participant before enrolling them in the study. This study complied with the Declaration of Helsinki.

Data analysis

The data entry was done in a Microsoft Excel spreadsheet, and the final analysis was done using IBM SPSS Statistics for Windows, Version 25.0 (Released 2017; IBM Corp., Armonk, NY, USA). Categorical variables were represented in numbers and percentages (%). Quantitative data having a normal distribution were presented as mean ± SD. The Shapiro-Wilk test was used to determine the normality of the distribution of vitamin D and HDL cholesterol levels. A chi-squared test was used to compare unpaired proportions like vitamin D and gender. A one-way ANOVA was used to compare the mean serum vitamin D levels in different seasons. The Kruskal-Wallis test was used to make group comparisons between vitamin D levels across different groups of disease activity. Bivariate variables like DAS28 ESR and serum vitamin D level, DAS28 ESR and HDL cholesterol level, and DAS28 ESR and non-HDL cholesterol level, which were not normally distributed, were assessed using Spearman’s correlation coefficient. A p-value ≤0.05 was considered statistically significant.

## Results

The study had 80 participants, the majority (34; 42.5%) of whom belonged to the age group of 35-43 years, while 17 (21.3%) were in the age group of 44-52 years. The oldest participant was 61 years old. Over half, 58 (72.5%) were females. A total of 37 (46.3%) presented with symptoms suggestive of RA in winter, 26 (32.5%) in spring/autumn, and 17 (21.3%) in summer. In winter, the highest number of patients (16; 20%) was reported in the month of January. A total of 66 (82.5%) showed RF positivity, 31 (38.8%) were anti-CCP positive, and nine (11.3%) were CRP positive. The mean ESR was 17.28 ± 6.93 mm/hour. The mean hemoglobin was 11.68 ± 1.48 gm/dl, and the mean total leukocyte count was 5,876 ± 1,634.3 mm³. The mean random blood sugar was 100.1 ± 14 mg/dl. The mean DAS28 ESR score was 3.81 ± 1.62, of which the majority (47; 58.8%) had moderate disease activity, 23 (28.8%) had severe, and 10 (12.5%) had low disease activity (Table 1).

**Table 1:** Baseline demographic and clinical features Anti-CCP, anti-citrullinated cyclic peptide; DAS28 ESR, Disease Activity Score 28 Erythrocyte Sedimentation Rate; RF, rheumatoid factor

Variable	Number	Frequency (%), N = 80
Age group		
17-25	4	5%
26-34	17	21.30%
35-43	34	42.50%
44-52	17	21.30%
>52	8	10%
Gender		
Female	58	72.50%
Male	22	27.50%
Season of presentation		
Summer	17	21.30%
Winter	37	46.30%
Others	26	32.50%
RF positivity		
Positive	66	82.50%
Negative	14	17.50%
Anti-CCP positivity		
Positive	31	38.80%
Negative	49	61.30%
CRP positivity		
Positive	9	11.30%
Negative	71	88.80%
DAS 38 ESR (disease activity)		
High	23	28.80%
Moderate	47	58.80%
Low	10	12.50%
Serum vitamin D levels		
Deficient	38	47.50%
Insufficient	23	28.80%
Normal	19	23.80%
Total cholesterol		
Borderline high	23	28.80%
High	7	8.80%
Normal	50	62.50%

**Figure 1:**
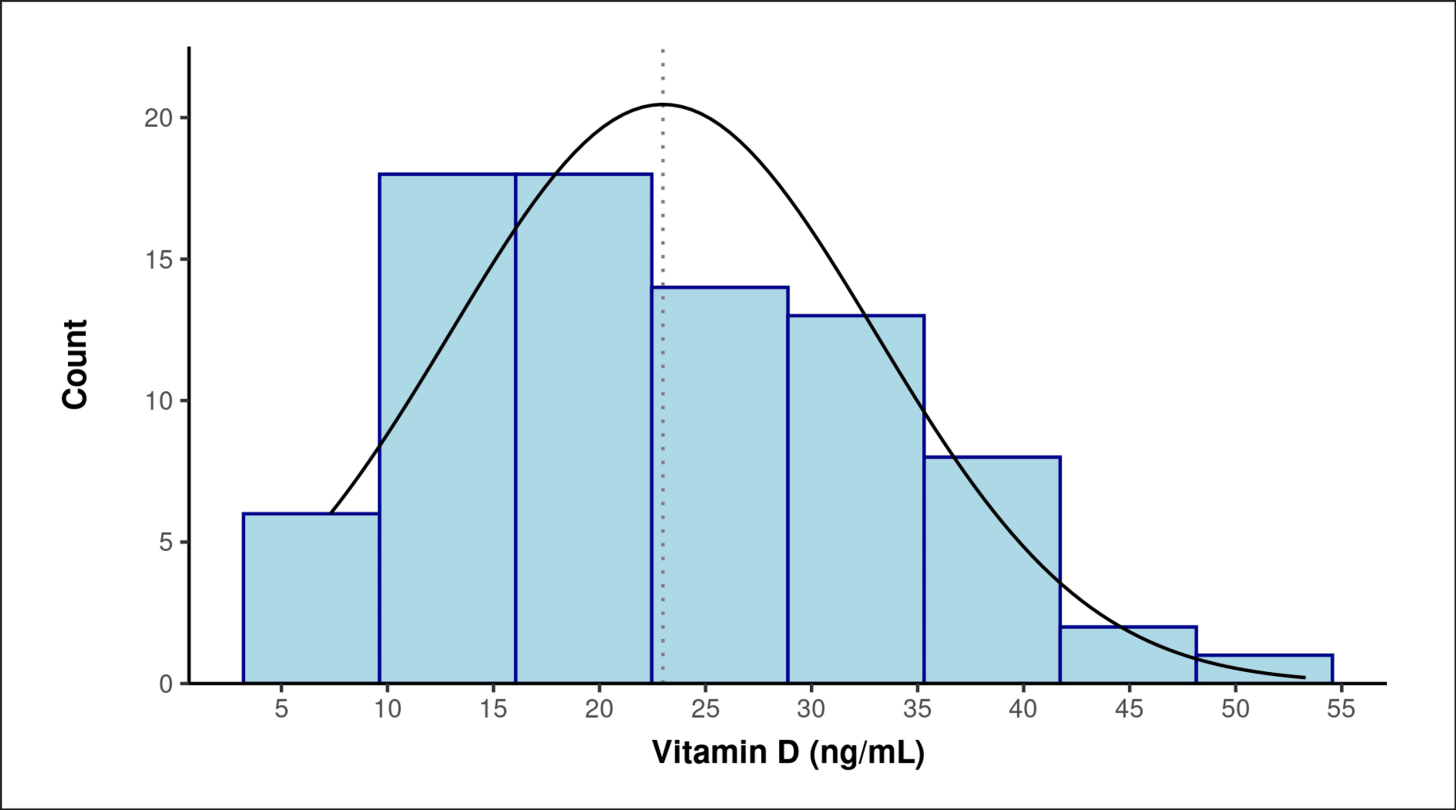
Distribution of serum 25-OH vitamin D level (ng/ml) in RA participants The mean SD of vitamin D (ng/mL) was 22.98 (10.01). The vitamin D (ng/mL) ranged from 7.3 to 53.3. The skewness of the data was 0.53, and it suggested that the data were positively skewed, thus implying they were not normally distributed. There appeared to be only one mode/peak in the data, thus making them unimodal. RA, rheumatoid arthritis

The mean value of 25-OH vitamin D was found to be 22.98 ± 10.01 ng/ml, with values ranging from 7.33-52.3 ng/ml (Table 2, Figure 1). Almost half of the participants (38; 47.5%) were vitamin D deficient, 23 (28.8%) had insufficient vitamin D levels, and the rest (19; 23.8%) had normal serum vitamin D levels (Figure 1).

**Table 2:** Serum biomarker levels and DAS28 ESR score of study participants DAS28 ESR, Disease Activity Score 28 Erythrocyte Sedimentation Rate; HDL, high-density lipoprotein

Serum biomarker	Mean	SD	Minimum	Maximum	Median
Serum 25-OH vitamin D level (ng/ml)	22.9888	10.0148	7.33	53.3	21.9
Total cholesterol (mg/dl)	196.4875	25.7923	160	255	191
Serum HDL (mg/dl)	42.3	7.228	28	59	42
Non-HDL cholesterol (mg/dl)	154.1875	30.158	104	223	149.5
DAS28 ESR score	3.8198	1.1905	1.62	6.33	3.71

In comparison, out of 58 females in the study, 28 (48.3%) were vitamin D deficient, whereas 10 (45.5%) of 23 male participants were vitamin D deficient (Table 3). No statistically significant gender-based differences were noted between serum vitamin D levels (p = 0.596, chi-square: 1.0347).

**Table 3:** Association of 25-OH vitamin D serum levels (ng/ml) with gender A chi-squared test was used to explore the association between gender and 25-OH vitamin D. There was no significant difference among the various groups in terms of distribution of vitamin D (χ2 = 1.035, p = 0.596).

Vitamin D	Gender			Chi-squared test	
	Male	Female	Total	χ^2^	p-value
Normal	4 (18.2%)	15 (25.9%)	19 (23.8%)	1.035	0.596
Insufficient	8 (36.4%)	15 (25.9%)	23 (28.7%)		
Deficient	10 (45.5%)	28 (48.3%)	38 (47.5%)		
Total	22 (100.0%)	58 (100.0%)	80 (100.0%)		

**Figure 2:**
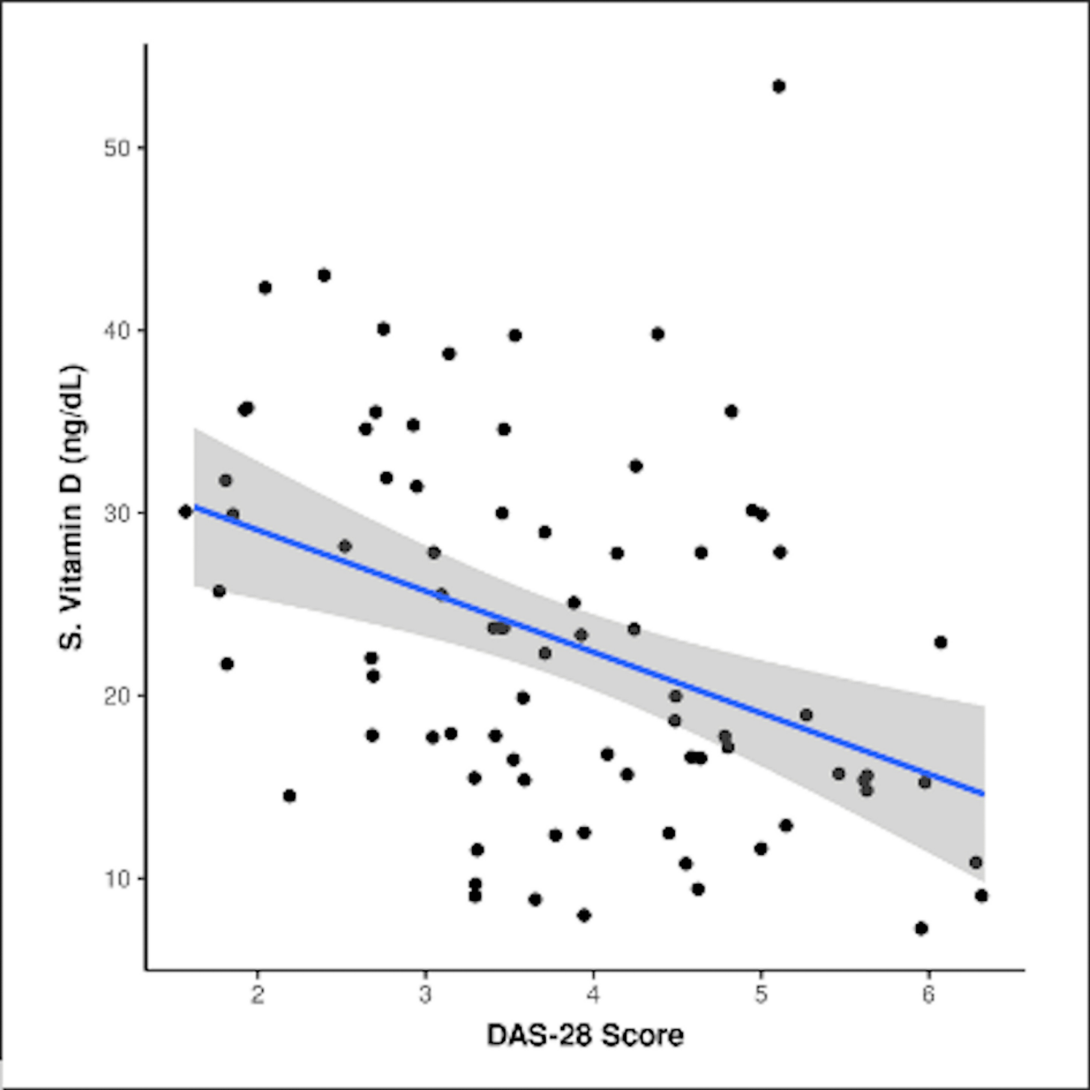
Correlation between DAS28 ESR and serum vitamin D level The preceding scatterplot depicts the correlation between the DAS28 score and serum vitamin D level (ng/ml). Individual points represent individual cases. The blue trendline represents the general trend of correlation between the two variables. The shaded gray area represents the 95% confidence interval of this trendline. Nonparametric tests (Spearman correlation) were used to explore the correlation between the two variables because at least one of the variables was not normally distributed. The value of R was -0.428. Although technically a negative correlation, a relationship was found between DAS28 ESR and vitamin D level. R 2, the coefficient of determination, was 0.1578, p = 0.00026 (p < 0.05), suggesting higher disease activity with lower vitamin D levels. The result is significant at p < 0.05. X-axis: DAS28 ESR; Y-axis: vitamin D (ng/ml) DAS28 ESR, Disease Activity Score 28 Erythrocyte Sedimentation Rate

On application of ANOVA, a statistically significant difference was seen in the serum vitamin D levels in summer and other seasons (p < 0.05). In the summer season, the mean level of vitamin D was 28 ± 8 ng/ml; in winter, it was 20.13 ± 10 ng/ml, and 23.26 ± 9.1 ng/ml in other seasons. The mean value of serum vitamin D (ng/ml) in those with high DAS28 ESR was 15.56 ± 5.69, 21.31 ± 9.95 in those with moderate disease activity, and 28.37 ± 8.02 in those with low disease activity. An inverse correlation was found between DAS 28 and serum vitamin D; with the value of R being -0.428, R 2, the coefficient of determination was 0.1578, p = 0.00026 (p < 0.05), suggesting higher disease activity with lower vitamin D levels (Figure 2). For every one-point increase in the DAS28 ESR score, the serum vitamin D (ng/dl) decreased by 3.34. Conversely, for every 1 ng/ml decrease in serum vitamin D (ng/ml), the DAS28 ESR score decreased by 0.05.

**Figure 3:**
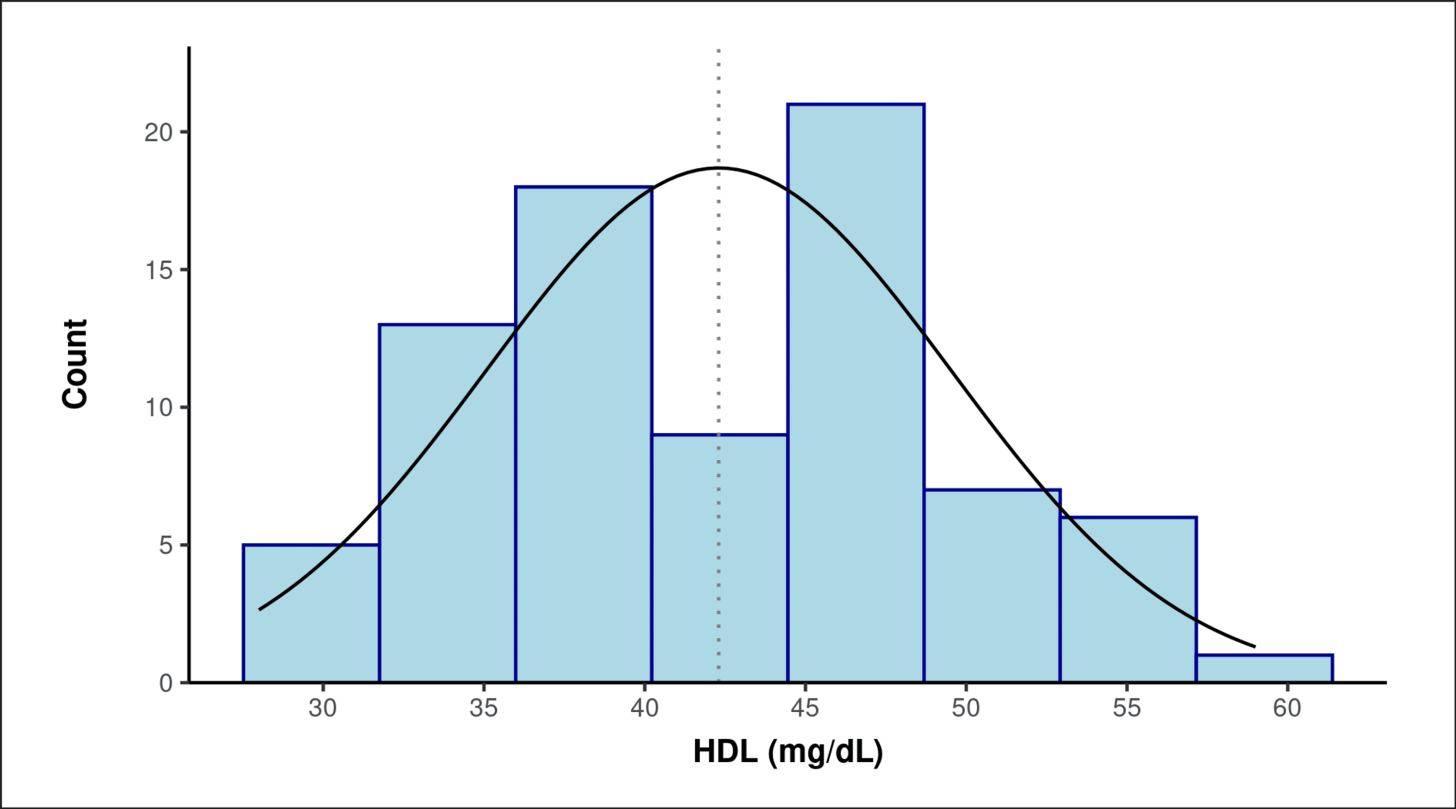
Distribution of serum HDL cholesterol level (mg/dl) in the study population The mean SD of HDL (mg/dL) was 42.30 (7.23). The median IQR of HDL (mg/dL) was 42.00 (37.45-47.33). The HDL (mg/dL) ranged from 28 to 59. The skewness of the data was 0.09, and it suggested that the data were not skewed, thus suggesting they were normally distributed. There appeared to be more than one mode/peak in the data, thus making them multimodal. HDL, high-density lipoprotein

The mean total cholesterol level was 196.48 ± 25.79 mg/dl, with 23 (28.8%) having borderline high, seven (8.8%) having high, and the majority, 50 (62.5%) having normal serum levels. The mean HDL cholesterol was 42.3 ± 7.23 mg/dl with values of 28-59 mg/dl (Table 2, Figure 3). Of patients aged 35-43 years, 27 (79.4%) had a low level of serum HDL cholesterol.

**Figure 4:**
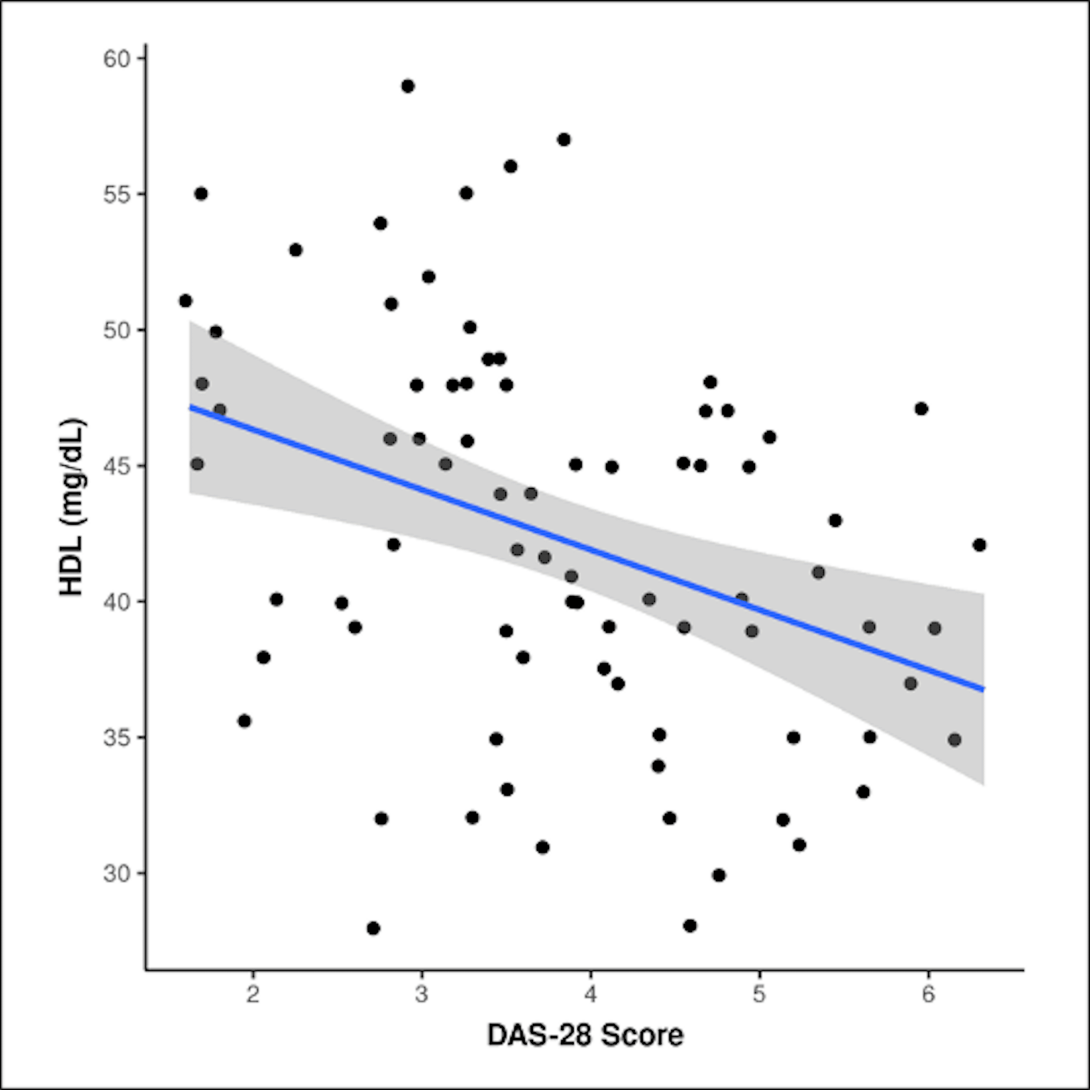
Correlation between DAS28 ESR and serum HDL cholesterol level (mg/dl) The preceding scatterplot depicts the correlation between the DAS28 ESR score and HDL cholesterol (mg/dl) level. Individual points represent individual cases. The blue trendline represents the general trend of correlation between the two variables. The shaded gray area represents the 95% confidence interval of this trendline. Nonparametric tests (Spearman’s correlation) were used to explore the correlation between the two variables because at least one of the variables was not normally distributed. The value of R was -0.3972. Although technically a negative correlation, a relationship was found between DAS28 and HDL. The value of R2, the coefficient of determination, was 0.1578. The p-value was 0.000349. The result was significant at p < 0.05. X-axis: DAS 28, Y-axis: HDL cholesterol DAS28 ESR, Disease Activity Score 28 Erythrocyte Sedimentation Rate; HDL, high-density lipoprotein

In more than two-thirds, 47 (81%) of female RA participants, serum HDL cholesterol level was abnormal. There was a significant difference between both genders in terms of distribution of HDL (χ2 = 9.857, p = 0.002). The mean HDL cholesterol in those with DAS28 ESR high disease activity score was 38.08 ± 4.62 mg/dl, 41.38 ± 6.77 mg/dl in those with moderate disease activity, and 46.07 ± 8.63 mg/dl in the low disease activity group. Also, an inverse relationship was established between HDL cholesterol and DAS28 ESR scores. A negative correlation was seen where the value of R was -0.3975, the R2 coefficient of determination was 0.1578, and the p-value was p-0.00034 (p < 0.05), signifying higher disease activity scores with lower HDL levels (Figure 4). For every one-point increase in the DAS28 ESR score, the HDL cholesterol (mg/dl) decreased by 2.21. Conversely, for every 1 mg/dl increase in serum HDL cholesterol, the DAS28 ESR score decreased by 0.06. A positive correlation was seen between non-HDL cholesterol and the DAS28 ESR score.

## Discussion

RA is defined as a chronic, systemic, autoimmune inflammatory disease with an estimated Indian prevalence of 0.75%. It is associated with disability, reduced quality of life, and premature mortality. RA is associated with fatigue and pain, which affect different domains of personal existence such as social relationships, vocational performance, and psychological well-being [9]. Therefore, the aim of treatment is to suppress inflammation and prevent further joint damage. Studies have shown that vitamin D exerts immune modulation, and its deficiency may lead to synovial destruction and increased disease activity quantified by scores such as the DAS 28 score [5,8,10]. It is also associated with accelerated atherogenesis and plaque formation, leading to cardiovascular mortality. HDL cholesterol, in addition to being anti-atherogenic, is anti-inflammatory and antioxidant in nature. The lipid goal in RA is not only to reduce cholesterol, triglyceride, and LDL cholesterol levels but also to improve HDL cholesterol levels [6]. Hence, we tried to establish the association of serum vitamin D and HDL levels with disease activity in RA using DAS28 ESR, which is extensively validated and a superior tool for monitoring disease activity.

Our study had 80 participants, of whom 34 (42.5%) were aged 35-43 years and 58 (72.5%) were females. In comparison to published studies, these participants had an earlier age of onset. Even though the commonest age of onset is the fifth decade, symptoms can start anytime between the third and fifth decade. Alpízar-Rodríguez et al. showed that the age of onset and disease manifestations were geographically variable [11]. Also, it was seen in his study that Asians had a female preponderance and an earlier age of onset. Female predominance can be explained by differences in sex hormones, as shown by Alpízar-Rodríguez et al. and Abdulsatar et al. [11,12]. This is because estrogens exert a pro-inflammatory effect, whereas androgens have an anti-inflammatory effect. Further, in RA, the conversion of androgens to estrogens is increased by inflammatory mediators, which stimulate aromatase activity in extra-gonadal tissues [11].

Over a third of participants, 37 (46.3%) had presented to the clinic during the winter season, and in particular, 16 (20%) had reported symptoms in the month of January, as in the findings of Feldthusen et al. of Sweden, who found that patients with RA had greater fatigue symptoms in winter, with the highest fatigue ratings in January [13]. Also, they showed that patients with RA reported the greatest fatigue between the months of September and January [13].

The mean ESR was 17.2 ± 6.93 mm AEFH, and the mean hemoglobin level was 11.68 ± 8 gm/dl. ESR is influenced by factors such as age, gender, levels of fibrinogen, immunoglobulins, RF positivity, and anemia. Two-thirds of the participants, 66 (82.5%) were RF positive, 31 (38.8%) were anti-CCP positive, and 49 (61.3%) were anti-CCP negative. Dubucquoi et al. showed that anti-CCP had 85% sensitivity and 90.9% specificity for RA [14]. This discrepancy can be explained by the fact that anti-CCP is present in only two-thirds of patients with RA and is associated with joint-erosive changes and extra-articular manifestations. Further, the presence of smoking and periodontitis increases the citrullination of collagen and α-enolase, affecting its positivity rate [15]. Also, the majority of our study population comprised females who were nonsmokers. The lower positivity rate for anti-CCP may be accounted for by the different genetic backgrounds, as shown by Guo et al. [16]. Recent studies have shown a genetic predisposition in those with anti-CCP-positive RA. Genes encoding HLA-DR, especially HLA-DR1 and HLA-DR4, also known as “shared epitopes,” are implicated. Further, a novel protein, known as tyrosine phosphatase non-receptor type 22, has gained attention because of polymorphisms associated with anti-CCP in different ethnicities resulting in variable phenotypes [17].

The mean DAS28 ESR score was 3.81 ± 1.19, with values ranging between 1.62 and 6.33, and the majority, 47 (58.8%), had moderate disease activity as in the findings of Panchal et al. of India [18].

The mean 25-OH vitamin D was 22.98 ± 10.01 ng/ml, and 28 (48.3%) were vitamin D deficient. The findings were similar to the other Indian studies done by Marwaha et al., Bose et al., and Handa et al. [1,19,20]. Factors such as poor nutrition, cultural traits, and low sun exposure can account for these findings. The mean serum calcium level was 8.22 mg/dl after correction of hypoalbuminemia, consistent with a normal physiological response to a diminished vitamin D level. Also, interseasonal variation of vitamin D levels was noted, with vitamin D being 28.49 ± 8 ng/ml in summer and lower values of 20 ± 10 ng/ml being seen during winter (p < 0.013). This is in line with the findings of Klingberg et al. of Sweden, where levels of vitamin D varied with the intensity of UV-B irradiation [21].

Serum vitamin D levels and DAS28 ESR scores were found to have an inverse relationship, which meant that with lower levels of vitamin D, DAS28 ESR scores increased. This was also reported by Kostoglou-Athanassiou et al., Bose et al., and Lee and Bae [5,19,22]. Vitamin D supplementation can be given as an immunomodulator rather than complementary to steroids in all cases. This can additionally help in pain relief and the prevention of osteoporosis.

RA patients have accelerated atherosclerosis due to widespread systemic inflammation. The lipid profile may paradoxically appear less atherogenic. The mean HDL cholesterol level was 42.3 ± 7.23 mg/dl, with values ranging from 28 to 59 mg/dl. We found a negative correlation between low serum HDL and high DAS28 ESR scores (p < 0.05). This finding was also seen in various studies, such as that of Ostojic and Bartolovic and Hadda et al. of North India [23,24]. In particular, in the RA population of North India, low HDL cholesterol is the most common abnormality. That HDL cholesterol is atheroprotective is a misconception. HDL cholesterol size and its CEC become deranged in systemic inflammation [25]. Further, the total cholesterol was 196/48 ± 25.79 mg/dl in our study, which is borderline high. Also, among the disease-modifying drugs in RA, tumor necrosis factor-α inhibitors and Janus kinase inhibitors paradoxically increase LDL cholesterol levels [26]. Statins have pleiotropic effects beyond their lipid-lowering activities, which include anti-inflammatory, antioxidant, and anti-apoptotic effects [27]. Thus, lipid-lowering therapy with statins in RA may be beneficial in reducing cardiovascular morbidity.

This study did not have a control group, and there was a lower number of male subjects. Therefore, this study does not give us the optimal vitamin D cutoff point by gender. Due to the paucity of studies on exact prevalence data of vitamin D status in Eastern India, a true assessment of vitamin D deficiency is not possible. Factors such as body mass index, parathyroid hormone levels, and dietary factors may be possible confounders in the assessment of vitamin D status in the study.

## Conclusions

There are low serum levels of vitamin D and HDL cholesterol in RA. Deficiency of both may contribute to the worsening and progression of disease activity. In addition to other conventional biomarkers, 25-OH vitamin D and HDL cholesterol serum levels may be gauged routinely to monitor disease activity in RA. Vitamin D supplementation in recommended doses may be protective in RA. It may also help in pain relief and fatigue. RA is associated with an unfavorable lipid profile. Dyslipidemia management is equally important to reduce cardiovascular morbidity in RA. Large sample studies on the cholesterol efflux and antioxidant properties of HDL cholesterol are warranted.
